# Moderate Dealumination of Zeolites via Chelation to Facilitate Pt Anchoring and Toluene Removal

**DOI:** 10.3390/toxics13090737

**Published:** 2025-08-31

**Authors:** Wenqi He, Zhipeng Qie, Huaizhong Xiang, Hassan Alhassawi

**Affiliations:** 1 College of Mechanical and Energy Engineering, Beijing University of Technology, Beijing 100124, China; hewenqi@emails.bjut.edu.cn; 2 Chongqing Research Institute of Beijing University of Technology, Chongqing 401121, China; 3 Department of Chemistry, Queen Mary University of London, London E1 4NS, UK; h.xiang@qmul.ac.uk; 4 Department of Chemical Engineering, The University of Manchester, Manchester M13 9PL, UK; hassan.alhassawi@manchester.ac.uk

**Keywords:** zeolite, dealumination, chelation, catalytic oxidation, toluene

## Abstract

Zeolites are promising materials for volatile organic compound (VOC) adsorption and catalytic oxidation, where tuning their structure via defect engineering can enhance adsorption capacity and active metal dispersion. In this study, a concentration-sensitive chelation strategy using diethylenetriaminepentaacetic acid (DTPA) was developed to achieve moderate dealumination for Beta and Y zeolites. For Y zeolite, 0.1 M DTPA treatment increased the toluene adsorption capacity from 59 to 110 mg/g. After platinum (Pt) loading, both DTPA-modified Beta- and Y-based catalysts showed improved toluene oxidation efficiency compared to their unmodified counterparts. Remarkably, the Y-DTPA-0.01-Pt catalyst achieved 90% toluene conversion at 150 °C with CO_2_ selectivity above 90%. DRIFTS and H_2_-TPR results confirmed that moderate dealumination by DTPA generated silanol defects in zeolite Y that strongly anchored Pt^2+^ in a highly dispersed form and suppressed PtO formation. Severe dealumination using 0.1 M DTPA created larger defects that favored the aggregation of Pt^0^ clusters whilst causing significant loss in the micropores, thus reducing the Pt loading content and catalytic activity. This work demonstrates a simple and effective approach to optimize zeolite-based catalysts by controlling defect formation through controllable chelation, offering new insights into VOC abatement via tailored support design.

## 1. Introduction

Volatile organic compounds (VOCs), including aromatic hydrocarbons such as toluene, are common constituents of industrial flue gases and vehicular emissions. Their toxicity, carcinogenicity, and contributions to ozone formation and secondary particulate matter pose serious risks to both human health and the environment [1,2]. As such, the efficient abatement of VOCs has received significant attention. Among existing technologies, the combination of adsorption and catalytic oxidation is widely regarded as one of the most effective approaches [3,4]. Porous adsorbents are able to capture VOCs under ambient conditions. Catalytic oxidation, on the other hand, converts VOCs into non-toxic products such as CO_2_ and H_2_O at 100–350 °C, typically using noble metal or metal oxide catalysts [5,6]. This strategy demands materials with high adsorption capacity, catalytic efficiency, and thermal stability.

Zeolites have emerged as highly promising materials, serving simultaneously as adsorbents and catalyst supports for the mitigation of gaseous pollutants [7]. Their high surface area, tunable framework composition, well-developed microporosity, and superior hydrothermal stability enable effective VOC capture and facilitate the uniform dispersion of active metal species such as Pt and Pd [8,9]. Among all zeolites, Beta and Y possess relatively large-pore, three-dimensional frameworks (12 MR) that allow faster molecular diffusion than ZSM-5 and layered nanoclays such as bentonite. Y zeolite usually has a relatively low Si/Al ratio (e.g., 2–3), while that of the Beta series is higher and tunable, enabling flexible control over framework polarity and metal–support interactions. Post-synthetic modifications, including steaming, acid leaching, and chelation, have been explored to tailor the framework polarity and introduce mesoporosity, thereby enhancing the adsorption performance and metal–support interaction [10]. For instance, Amel et al. [11] demonstrated that dealumination of zeolite omega via acidic, basic, and hydrothermal treatments significantly increased its toluene adsorption capacity by 68% relative to the parent sample (high to 137.18 mg/g). Similarly, framework defects generated through dealumination, particularly silanol nests, can act as anchoring sites for metal cations, promoting high dispersion and preventing aggregation. This makes Pt especially effective among noble metals for VOC oxidation, as its dispersion and catalytic efficiency can be maximized on defect-rich zeolite supports. Run et al. [12] found that Pt supported on dealuminated Beta zeolites exhibited enhanced dispersion and superior catalytic oxidation activity for toluene at lower temperatures compared to unmodified supports.

However, traditional dealumination methods often suffer from poor controllability. Uncontrolled or excessive removal of framework aluminum can lead to the formation of large vacancy clusters, collapse of microporous channels, and significant loss of surface area [13]. These structural degradations impede VOC diffusion and limit the number of accessible active sites, ultimately diminishing both adsorption and catalytic performance. In our previous work, we proposed a post-synthetic modification strategy involving bulky organic chelating agents such as ethylenediaminetetraacetic acid (EDTA) [14,15]. Owing to the steric hindrance of the chelating molecules and the spatial confinement imposed by zeolite micropores, dealumination could proceed in a more spatially restricted and mild fashion, thereby preserving the framework integrity. Nonetheless, properties of the framework defects generated by such treatments, and their interaction with supported metal species, lack of in-depth understanding.

In this study, a moderate dealumination strategy using the organic chelating agent diethylenetriaminepentaacetic acid (DTPA) is proposed to precisely tailor the defect environment of Beta and Y zeolites. The influence of this treatment on toluene adsorption performance was first investigated, followed by evaluation of the catalytic activity after Pt loading for toluene oxidation. To reveal the interaction between Pt species and the defect-rich zeolite frameworks, a combination of diffuse reflectance infrared Fourier transform spectroscopy (DRIFTS), X-ray photoelectron spectroscopy (XPS), and H_2_ temperature-programmed reduction (H_2_-TPR) analyses was employed. These techniques provided detailed insights into the oxidation states, dispersion behavior, and anchoring environments of Pt species, especially their interactions with silanol (Si–OH) groups. Based on the comprehensive characterization, the structure–performance relationships were elucidated, offering valuable guidance for defect engineering in zeolite-based VOC adsorbents and catalysts.

## 2. Experimental

### 2.1. Materials

Zeolite Beta (SAR = 12.5) and Y (SAR = 2.6) were purchased from Nankai Catalyst Co., Ltd. (Tianjin, China) Diethylenetriaminepentaacetic acid (DTPA, AR) was obtained from Merck (Beijing, China) and used as the chelating agent. Chloroplatinic acid hydrate solution (8 wt% in water) was supplied by Aladdin (Shanghai, China) and served as the platinum precursor. Toluene standard gas (420 ppm, balanced with N_2_) and O_2_ (99.999%) gas were purchased from Shuangquan Tianyuan Gas Co., Ltd. (Beijing, China).

### 2.2. Post Treatment of Zeolites

In the dealumination process, 2 g of zeolite (Beta or Y type) was dispersed in 30 mL of deionized water under three different conditions: without DTPA, with 0.01 M DTPA, and with 0.1 M DTPA, resulting in six sample groups. The solid–liquid mixtures were heated at 100 °C using oil bath for 15 min. Afterward, the mixtures were centrifuged at 5000 rpm to separate the solids from the supernatant. The collected solids were subsequently washed three times with deionized water until the pH of the washings approached neutral (pH ≈ 7). The resulting solids were subsequently dried overnight at 80 °C. The treated samples were denoted as X-parent, X-DTPA-0.01, or X-DTPA-0.1, where X denotes the zeolite type (Beta or Y). For Pt loading, 0.5 g of the pretreated zeolite was added to 30 mL of an aqueous solution containing 0.5 wt% chloroplatinic acid. The suspension was ultrasonicated for 4 h to ensure uniform dispersion and deposition of Pt species into zeolite. After ultrasonication, the solid was dried overnight at 80 °C, followed by calcination in a tube furnace (1100X, Kejing, Hefei, China) at 450 °C for 1 h with a ramp rate of 5 °C min^−1^. The resulting catalysts were denoted as X-parent-Pt, X-DTPA-0.01-Pt, and X-DTPA-0.1-Pt, where X represents the zeolite type (Beta or Y).

### 2.3. Characterizations

Pore structure properties of the samples were characterized by N_2_ adsorption–desorption at −196 °C using a Beishide analyzer (BSD-660, Beishide, Beijing, China). Prior to measurement, the samples were degassed at 300 °C for 5 h. X-ray diffraction (XRD) patterns were obtained using a diffractometer (Ultima IV, Rigaku, Tokyo, Japan) with Cu Kα radiation (λ = 1.5406 Å) at a scanning rate of 1 °/min. The relative crystallinity of the zeolites was determined based on a method described in our previous study [16]. Elemental composition was analyzed by X-ray fluorescence (XRF) spectroscopy on a spectrometer (Axios, PANalytical, Almelo, The Netherlands), and the Si/Al molar ratios (SAR) were calculated accordingly. Dynamic vapor sorption (DVS) experiments were performed using a commercial system (BSD-DVS, Beishide, Beijing, China). Prior to testing, 50 mg of zeolite was pretreated at 150 °C under a continuous N_2_ flow for 4 h. The adsorption experiments were then conducted with a toluene gas flow of 420 ppm at 400 mL/min. During adsorption, the zeolite samples were placed in small crucibles with a diameter of 1 cm, suspended inside a sealed chamber and continuously exposed to the toluene gas flow, The sample weight changes were monitored in real time using a high-precision balance with a resolution of 0.001 mg. the X-ray photoelectron spectroscopy (XPS) was carried out on a spectrometer (K-Alpha, Thermo Fisher Scientific, Waltham, MA, USA) equipped with a monochromatic Al Kα X-ray source (hν = 1486.6 eV) to analyze surface elemental states. Scanning electron microscopy (SEM) was performed using a ZEISS microscope (GeminiSEM 300, ZEISS, Oberkochen, Germany), which provides resolutions of 1.0 nm at 15 kV and 1.6 nm at 1 kV, with an accelerating voltage range of 0.02–30 kV. Energy-dispersive X-ray spectroscopy (EDS) mapping was conducted using a detector (SmartEDX, ZEISS, Oberkochen, Germany). Transmission electron microscopy (TEM) images were collected using an FEI microscope (Talos F200X G2, Thermo Fisher Scientific, Waltham, MA, USA). Fourier-transform infrared (FTIR) spectra were recorded on a spectrometer (iS20, Thermo Fisher Scientific, Waltham, MA, USA) in transmission mode with 32 scans per sample. Diffuse reflectance infrared Fourier transform spectroscopy (DRIFTS) measurements were conducted on a spectrometer (VERTEX 80v, Bruker, Billerica, MA, USA) in the range of 400–4000 cm^−1^ to probe surface functional groups. Hydrogen temperature-programmed reduction (H_2_-TPR) was performed using a Micromeriticschemisorption instrument (AutoChem II 2920, Micromeritics, Norcross, GA, USA). For each test, 20 mg of catalyst was loaded into a U-shaped quartz reactor, pretreated under a He flow (50 mL/min) at 150 °C for 1 h, then cooled to 50 °C. A 10% H_2_/Ar mixture was introduced, and the sample was heated to 800 °C at 10 °C/min. H_2_ consumption was monitored using a thermal conductivity detector (TCD) embedded in the chemisorption instrument. Similarly, NH_3_-TPD test was conducted using a Micromeritics chemisorption instrument (AutoChem II 2920, Micromeritics, Norcross, GA, USA) (ca. 50 mg sample, ramp rate 10 °C/min, He flow 30 cm^3^/min) to acquire the acidity properties of the zeolites.

### 2.4. Toluene Catalytic Oxidation

Catalytic oxidation of toluene was carried out in a self-assembled fixed-bed reactor system, as described in our previous publication [17]. In this study, the feed gas consisted of 420 ppm toluene, 20% O_2_, and the balance N_2_, with a total flow rate of 100 mL/min. A total of 0.1 g of catalyst was loaded into the reactor and mixed with 0.5 g of quartz sand (40–60 mesh) to minimize mass transfer limitations. The outlet concentration of toluene was monitored using a photoionization detector (PID, Kernuo, Shenzhen, China), while the CO_2_ concentration was measured using an infrared gas sensor (GT-1000, Kernuo, Shenzhen, China) with an accuracy of ±1% of the reading.

## 3. Results and Discussion

### 3.1. Toluene Adsorption on Dealuminated Zeolites

**Figure 1:**
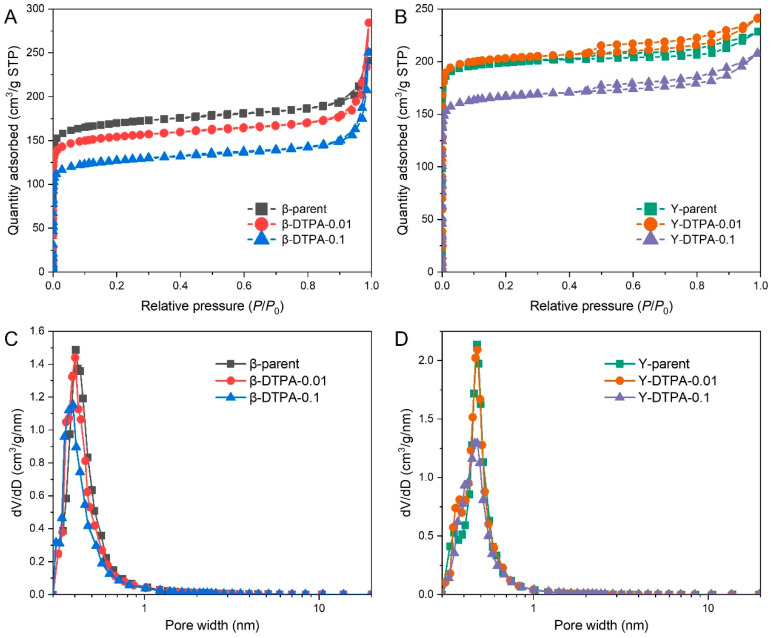
N_2_ adsorption–desorption isotherms measured at −196 °C for (**A**) Beta and (**B**) Y zeolites. DFT pore size distribution profiles of (**C**) Beta and (**D**) Y zeolites calculated from N_2_ physisorption data.

The N_2_ adsorption–desorption isotherms of Beta and Y zeolites before and after chelation treatment are shown in Figure 1A,B. The isotherms of Beta zeolites are close to type I, with a distinct increase in N_2_ uptake at high relative pressures (*P*/*P*_0_ > 0.9), indicating the coexistence of micropores and intercrystalline mesopores. This is further supported by the pore hierarchy (i.e., *V*_mic_/*V*_t_ ranging from 0.47 to 0.63) listed in Table 1. After DTPA treatment, both β-DTPA-0.01 and β-DTPA-0.1 samples show a significant decrease in N_2_ uptake at *P*/*P*_0_ < 0.01, although the isotherm type remains unchanged. This suggests that the loss of microporosity arises from partial framework destruction and the formation of amorphous species, which can probably migrate into the channels and block the micropores during DTPA chelation. Similar pore-blocking effects have also been reported when amorphous species are deposited within zeolite channels [18]. Specifically, the BET surface area of the parent Beta zeolite decreases from 670 m^2^/g to 609 m^2^/g and 496 m^2^/g, while the micropore volume drops from 0.24 cm^3^/g to 0.23 cm^3^/g and 0.18 cm^3^/g, respectively. The corresponding DFT pore size distribution (PSD) profiles of the Beta zeolites, shown in Figure 1C, also confirm the reduced microporosity centered around approximately 0.6 nm. The Y zeolites exhibit typical type I isotherms, with a small hysteresis loop appearing at *P*/*P*_0_ > 0.4, indicating that microporosity is dominant with a small amount of mesopores. This is consistent with their high pore hierarchy values (*V*_mic_/*V*_t_ of 0.76 to 0.86). After treatment with 0.01 M DTPA, a slight increase in N_2_ uptake is observed, and the surface area increases from 810 m^2^/g to 827 m^2^/g. This suggests that mild chelation with DTPA may help remove pore-blocking EFAl species with minimal damage to the FAU zeolite framework. However, when the DTPA concentration is increased to 0.1 M, a notable decrease is observed in both surface area and micropore volume, from 810 m^2^/g to 670 m^2^/g and from 0.30 cm^3^/g to 0.25 cm^3^/g, respectively. These changes in porosity are also supported by the corresponding DFT PSD data in Figure 1D.

**Figure 2:**
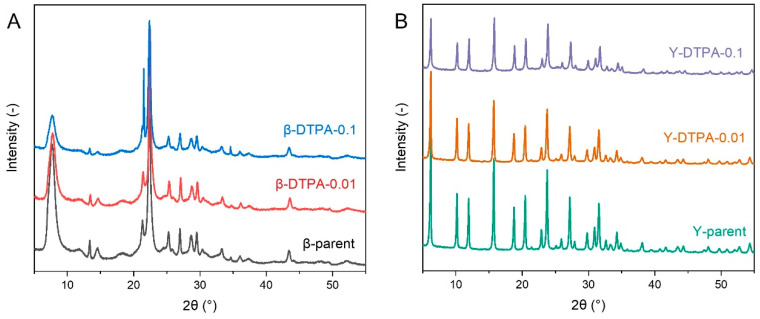
XRD patterns of the (**A**) Beta and (**B**) Y zeolites.

The structural stability of Beta and Y zeolites is differently affected by DTPA treatment, as shown by the XRD patterns of the parent and modified zeolites in Figure 2. As for the Beta zeolite, the diffraction peak positions in Figure 2A remain unchanged after treatment with 0.01 M and 0.1 M DTPA, indicating that the BEA-type framework is preserved. However, a noticeable decrease in peak intensity is observed, with the relative crystallinity declining from 100% in the parent Beta to 80% in β-DTPA-0.01 and 62% in β-DTPA-0.1. This suggests that even at a low DTPA concentration of 0.01 M, distinctive removal of framework aluminum occurs, evidenced by the increase in SAR from 12.5 to 17.5, accompanied by a decrease in the total acid amount from 1.27 mmol/g to 0.84 mmol/g (Table S1). The extent of structural degradation intensifies with increasing DTPA concentration to 0.1 M (SAR further increases to 23.5). Differently, as shown in Figure 2B, the Y zeolite treated with 0.01 M DTPA maintains a relative crystallinity above 90%, while treatment with 0.1 M DTPA leads to a more significant reduction to 55%. Even after 0.1 M DTPA treatment, the Si/Al ratio of the Y zeolite increases only to 5.7, which is consistent with previous reports on chelation treatment of Y zeolite [19]. All these changes in crystallinity align with the observed loss of pore volume after DTPA treatment. The differing sensitivity to DTPA-induced dealumination between Beta and Y zeolites can be attributed to their different framework topologies. Beta zeolite features a three-dimensional, fully connected 12-membered ring (12-MR) channel system, which allows partial diffusion of the bulky DTPA molecules into the pore network even at low concentrations. This leads to more extensive structural degradation in Beta. In contrast, Y zeolite has a cage-like structure composed of supercages interconnected by relatively narrow channels, which sterically hinder the diffusion of DTPA molecules, thereby limiting the extent of dealumination within the Y zeolite framework.

**Figure 3:**
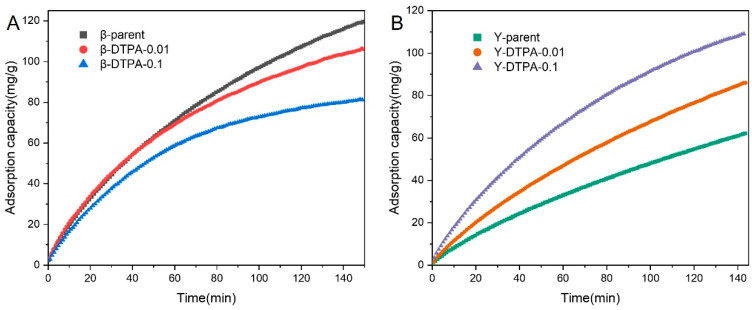
Dynamic adsorption curves of toluene (420 ppm) on the (**A**) Beta and (**B**) Y zeolites.

Figure 3A,B present the dynamic toluene (420 ppm) adsorption curves, highlighting the contrasting behavior of Beta and Y zeolites. For the Beta zeolite, the parent sample exhibits a high toluene uptake capacity of 120 mg/g within 150 min. After treatment with 0.01 M DTPA, the capacity decreases to 106 mg/g, and further drops to 78 mg/g at 0.1 M. This decrease indicates that the loss of porosity caused by DTPA-induced dealumination has a strong negative impact on toluene capture performance. In contrast, the parent Y zeolite shows a much lower adsorption capacity of 59 mg/g, significantly lower than any of the Beta samples. This can be attributed to its much lower SAR (2.6) compared to that of Beta (12.5), resulting in a framework with higher polarity, which limits its affinity toward toluene molecules with weak polarity. After DTPA treatment, a clear enhancement in toluene adsorption capacity is observed: Y-DTPA-0.01 reaches 82 mg/g and Y-DTPA-0.1 further increases to 110 mg/g, even surpassing the modified Beta samples. Despite the reduction in surface area in dealuminated Y zeolites, the improved toluene adsorption capacity can be ascribed to more favorable framework chemistry, particularly the reduced polarity. Although the dynamic adsorption behavior within 150 min did not reach complete equilibrium, kinetic model fitting (using pseudo-first-order and pseudo-second-order models) was employed to provide an overall understanding of toluene adsorption on Beta and Y zeolites and to predict the saturated adsorption capacity. As shown in Figure S1 and Table 2, the pseudo-first-order model provides a significantly better fit (*R*^2^ > 0.995) compared to the pseudo-second-order model. This suggests that toluene uptake on these zeolites follows a physical adsorption mechanism rather than a chemical one [20]. Specifically, the pseudo-first-order rate constant *K*_1_ for toluene adsorption on Beta zeolites follows the trend: β-parent > β-DTPA-0.01 > β-DTPA-0.1, whereas for Y zeolites, the trend is reversed: Y-parent < Y-DTPA-0.01 < Y-DTPA-0.1. This trend in the pseudo-first-order constant *K*_1_ indicates that DTPA modification impairs the adsorption kinetics of Beta zeolites but significantly enhances those of Y zeolites, highlighting a more favorable kinetic response to dealumination in the latter.

Overall, mild treatment with low-concentration DTPA (0.01 M) effectively preserves the crystallinity and pore structure of both Y and Beta zeolites, while notably enhancing the toluene adsorption capacity and kinetics of Y zeolite.

### 3.2. Pt Loading and Toluene Catalytic Oxidation

The obtained Beta and Y zeolites were used as supports for Pt loading via an impregnation method (theoretical Pt content 0.5 wt%), and DTPA treatment was found to influence the Pt loading in a concentration-dependent manner. XPS analysis was conducted to determine the actual content and valence states of Pt species on the zeolites, as summarized in Table 3 (survey spectra are provided in Figure S2). In general, the actual Pt contents on Y zeolites were found to be higher than those on Beta zeolites, indicating that Y zeolite offers a more favorable environment for Pt dispersion, likely due to its relatively larger BET surface area. Interestingly, a consistent trend was observed across both zeolite types: treatment with 0.01 M DTPA resulted in higher Pt loading compared to the parent samples. For example, the Pt content increased from 0.18 at% on parent Beta to 0.21 at% on β-DTPA-0.01-Pt. This enhancement can be attributed to the removal of EFAl species from the zeolite pores, which facilitates better diffusion and stabilization of Pt species. However, when the DTPA concentration was increased to 0.1 M, the Pt loading on β-DTPA-0.1-Pt and Y-DTPA-0.1-Pt decreased to 0.13 at% and 0.34 at%, respectively, being even lower than those on their parent counterparts. This decrease in Pt loading content is due to the loss of available anchoring sites caused by the reduced surface area after a more severe dealumination.

**Figure 4:**
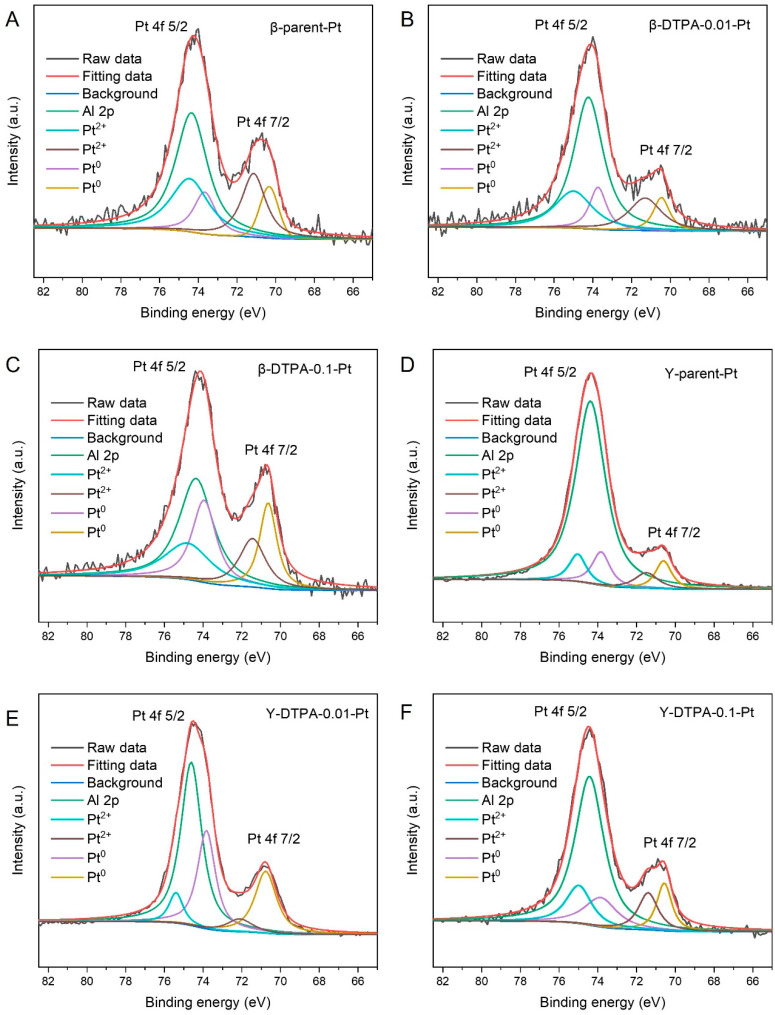
High resolution XPS Pt 4f spectra of (**A**) β-parent-Pt, (**B**) β-DTPA-0.01-Pt, (**C**) β-DTPA-0.1-Pt, (**D**) Y-parent-Pt, (**E**) Y-DTPA-0.01-Pt, (**F**) Y-DTPA-0.1-Pt.

Figure 4A–F show the high-resolution Pt 4f XPS spectra of all Pt-loaded zeolites. The spectra were deconvoluted into Pt^0^ and Pt^2+^ species, with the Pt 4f_7/2_ and Pt 4f_5/2_ peaks exhibiting a fixed splitting of approximately 3.3 eV in binding energy. Specifically, the binding energies for Pt^0^ are 71.2 eV (4f_7/2_) and 74.5 eV (4f_5/2_), while those for Pt^2+^ appear at 72.4 eV (4f_7/2_) and 75.7 eV (4f_5/2_). Notably, no peaks corresponding to Pt^4+^ species were observed in any sample. Additionally, the Al 2p signal at around 74.2 eV overlaps with the Pt 4f_5/2_ signals, which complicates the deconvolution of Pt species [21]. To quantitatively evaluate the valence states and redox properties of Pt species on the zeolites, the area ratio of Pt^0^ to Pt^2+^ was calculated and is listed in Table 3. In this study, the Pt^0^/Pt^2+^ ratios of β-parent-Pt and Y-parent-Pt are 0.47 and 0.69, respectively. After treatment of the zeolite supports with 0.01 M DTPA, the Pt^0^/Pt^2+^ ratios slightly increased to 0.50 for β-DTPA-0.01-Pt and 0.70 for Y-DTPA-0.01-Pt. When the DTPA concentration was increased to 0.1 M, the Pt^0^/Pt^2+^ ratios significantly increased to 1.14 for β-DTPA-0.1-Pt and 0.98 for Y-DTPA-0.1-Pt. These results indicate that treatment with 0.1 M DTPA substantially increases the proportion of metallic Pt^0^ on the zeolite surface, whereas the effect is negligible at the lower concentration of 0.01 M. The higher proportion of Pt^0^ is attributed to the formation of more Pt^0^ clusters or nanoparticles on the zeolite supports.

**Figure 5:**
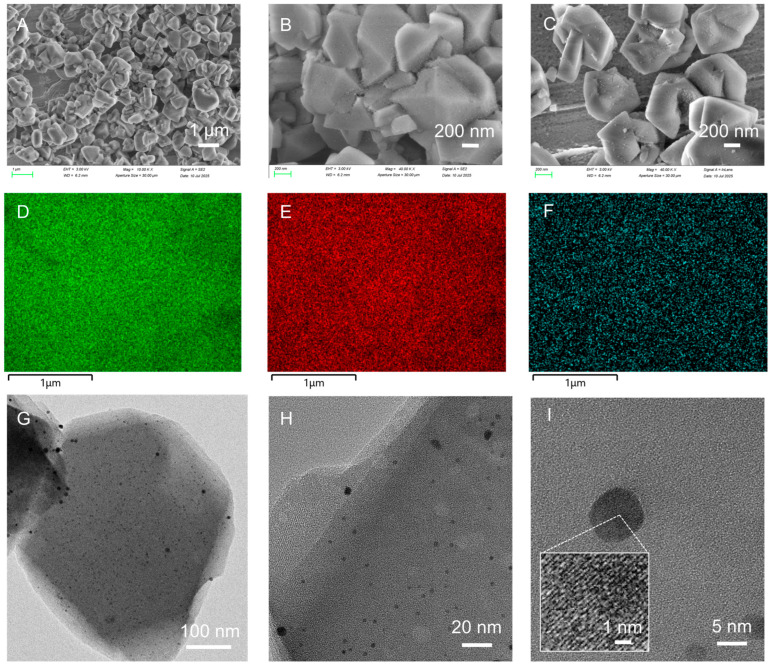
(**A**–**C**) SEM images of Y-DTPA-0.01-Pt at different magnifications. (**D**–**F**) EDS elemental mapping of Si, Al, and Pt, respectively. (**G**–**I**) High-resolution TEM images of Y-DTPA-0.01-Pt, with highlighting a single Pt particle showing lattice fringes (inset in Figure 5I).

**Figure 6:**
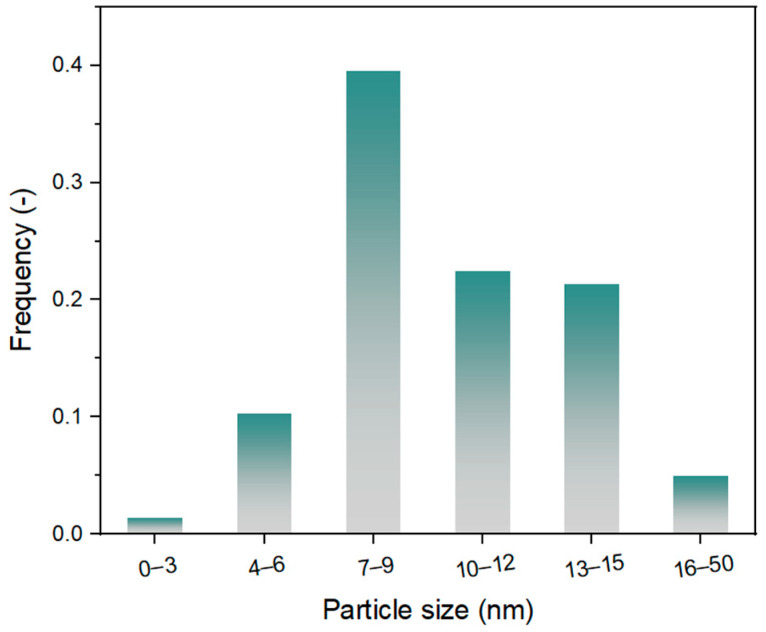
Particle size distribution of Pt loaded on Y-DTPA-0.01-Pt.

Based on the above analysis, sample Y-DTPA-0.01-Pt, which exhibits the highest Pt content along with a moderate Pt^0^/Pt^2+^ ratio, was selected as a representative for further discussion. SEM images of Y-DTPA-0.01-Pt are shown in Figure 5A–C. Figure 5A shows the typical aggregation characteristics of FAU crystals, with each crystal having geometric shapes ranging from hundreds of nanometers to microns. Higher-magnification images in Figure 5B,C reveal that the external surfaces of Y-DTPA-0.01-Pt remain smooth, with some nanosized particles observed on the surface, which are tentatively attributed to Pt nanoparticles introduced by impregnation. Figure 5D–F present SEM-EDS elemental maps of Si, Al, and Pt across the zeolite surface, showing that Pt species are uniformly distributed on the external surface of Y-DTPA-0.01-Pt. HRTEM images in Figure 5G illustrate that the internal framework of Y-DTPA-0.01-Pt remains dense and well-ordered, indicating that the mild 0.01 M DTPA treatment did not cause visible damage to the FAU zeolite microstructure. In addition, darker features at the nanometer scale are visible throughout the zeolite matrix, suggesting the presence of uniformly dispersed Pt nanoparticles. An HRTEM image in Figure 5H provides a clearer view of the Pt nanocrystals, form which the particle size distribution was extracted using threshold segmentation methods and is shown in Figure 6. It presents that the size of Pt nanoparticles loaded on Y-DTPA-0.01-Pt nearly follows a normal distribution, mostly within the size range of 7 to 9 nm. Although the frequency of particles in the 0–3 nm range is relatively low, some nano- or sub-nanometer Pt species (e.g., ultra-small clusters or single atoms) may not be able to be detected by the TEM with such a resolution. Figure 5I with a higher magnification shows lattice fringes with a spacing of 0.226 nm for the Pt nanoparticles, corresponding to the (111) plane of metallic Pt^0^ rather than Pt oxide, confirming the presence of Pt^0^ nanoparticles.

**Figure 7:**
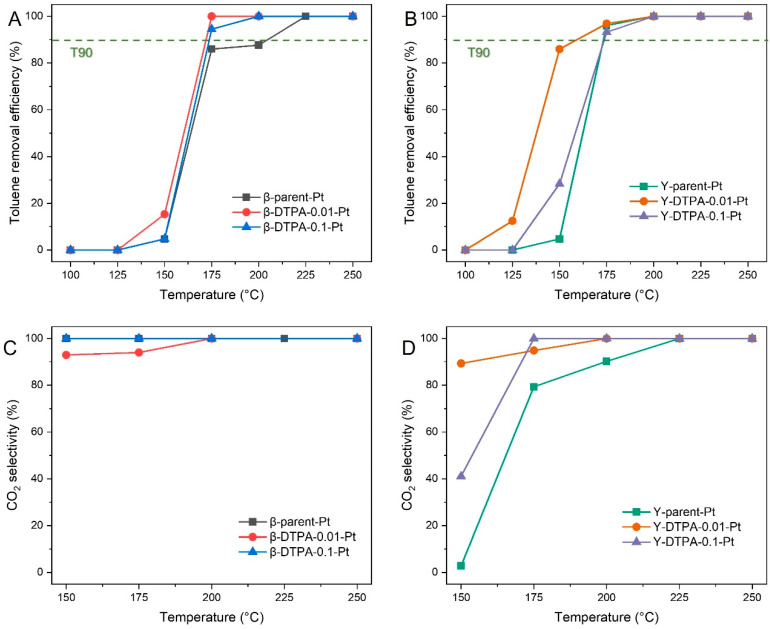
Catalytic oxidation performance of Pt-loaded zeolites toward toluene (420 ppm): (**A**,**B**) show the toluene conversion efficiency over Beta- and Y-type zeolites, respectively. (**C**,**D**) present the corresponding CO_2_ selectivity of the converted toluene for the Beta and Y series.

Figure 7A,B show the toluene catalytic oxidation efficiency over the Beta and Y series zeolite catalysts loaded with Pt, respectively. Overall, the Y-series catalysts require significantly lower temperatures for toluene oxidation compared to their Beta-series counterparts. For example, Y-DTPA-0.01-Pt achieves 90% toluene removal efficiency at 150 °C, while β-DTPA-0.01-Pt reaches only 15% at the same temperature. This difference can be supported by characterization results, which reveal higher Pt content and more developed micropores for the Y-series zeolites, providing a greater number of active sites for catalytic oxidation. As shown in Figure 7A, the T_90_ values (the temperature at which toluene removal efficiency is 90%) for the Beta-series catalysts follow the order: β-DTPA-0.01-Pt > β-DTPA-0.1-Pt > β-Parent-Pt. A similar trend is observed for the Y-series catalysts in Figure 7B, with the sequence Y-DTPA-0.01-Pt > Y-DTPA-0.1-Pt > Y-Parent-Pt. These results confirm that DTPA treatment of the supports enhances the catalytic performance of Pt-loaded zeolites for toluene oxidation. In addition, catalysts supported on zeolites treated with mild DTPA chelation (0.01 M) outperform those treated more severely (0.1 M). This can be tentatively attributed to a higher Pt loading content in Y-DTPA-0.01-Pt than Y-DTPA-0.1-Pt, making the active sites in the former is more sufficient than in the latter. Compared with conventionally post-treated zeolite catalysts, Beta-DTPA-0.01 obtained via chelation exhibits a higher specific surface area than the HNO_3_-treated sample (as listed in Table S2), while its Pt-loaded counterpart achieves a markedly lower T_90_ in toluene oxidation (ca. 160 °C vs. 190 °C). For Y zeolite, although Y-DTPA-0.01-Pt shows a T_90_ comparable to that of catalysts post-treated by NH_4_HF_2_ and NH_4_OH treatments (ca. 150 °C vs. 149 °C), the chelation strategy employed here avoids the use of fluorides, thereby mitigating potential environmental concerns [22].

Figure 7C,D illustrate the CO_2_ selectivity during toluene catalytic oxidation. As for the Beta series, all catalysts exhibit CO_2_ selectivity high to 99% at temperatures above 200 °C, indicating the nearly complete conversion from toluene into CO_2_ and H_2_O. At lower temperatures (150 and 175 °C), the selectivity decreases to approximately 92%, accompanied by the formation of partial oxidation intermediates such as benzaldehyde or benzoic acid. Among Y series samples, the Y-DTPA-0.01-Pt catalyst delivers the best overall performance in the Y series, achieving both the highest toluene conversion and excellent CO_2_ selectivity, i.e., exceeding 90% even at 150 °C and reaching complete oxidation above 200 °C. In comparison, Y-DTPA-0.1-Pt, which contains fewer Pt active sites, exhibits lower selectivity, and the untreated Y-parent-Pt performs also being worse than Y-DTPA-0.01-Pt. The superior performance of Y-DTPA-0.01-Pt can be attributed to its combination of the highest Pt loading content and a moderate Pt^0^/Pt^2+^ ratio, which together enhance both toluene oxidation and CO_2_ selectivity, confirming the effectiveness of moderate dealumination by DTPA imposed on zeolite support.

### 3.3. Mechanisms for Defect-Pt Interaction

**Figure 8:**
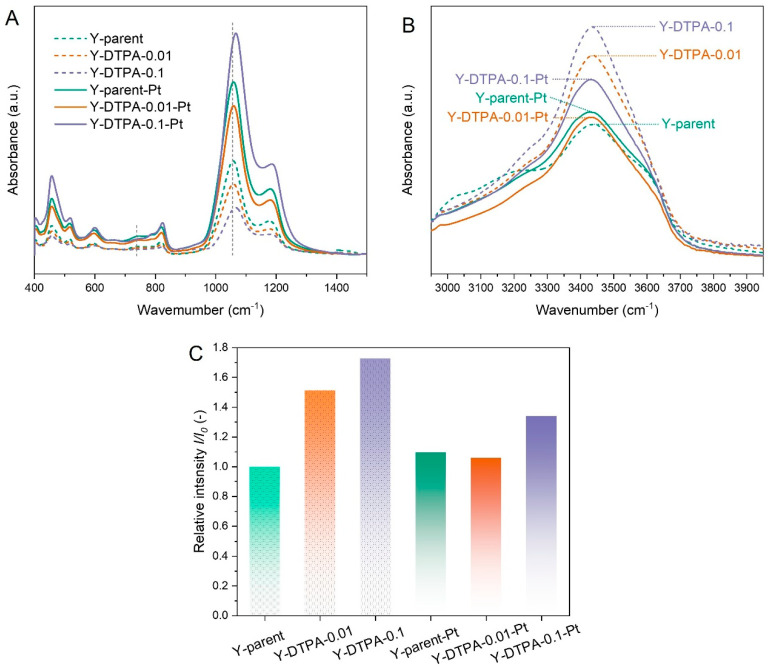
(**A**) FTIR spectra of Y zeolites and Pt-loaded Y zeolites in the range of 400–1500 cm^−1^. (**B**) DRIFTS spectra of Y zeolites and Pt-loaded Y zeolites in the range of 3000–4000 cm^−1^. (**C**) Relative intensity of hydroxyl groups (*I*/*I*_0_) derived from the DRIFTS data in Figure 8B.

Y-DTPA-0.01-Pt exhibits the highest catalytic performance for toluene oxidation among all samples, however, the specific state of Pt species on the zeolite support remains to be further elucidated. Figure 8 presents the FTIR spectra of Y zeolites before and after Pt loading. As shown in Figure 8A, in the wavenumber range of 400–1500 cm^−1^, all samples display a characteristic band at 717 cm^−1^, corresponding to the symmetric and asymmetric stretching vibrations of external linkages in double six-membered rings (6-MRs). This indicates that the fundamental FAU framework remains largely intact after DTPA treatment. However, the intensity of this band decreases after 0.01 M DTPA treatment, with a more significant reduction observed for the 0.1 M treated sample. In addition, a shift of the band around 1010 cm^−1^ (attributed to the internal asymmetric stretching vibrations of the framework) toward higher wavenumbers is observed after 0.1 M DTPA treatment. These spectral changes, in conjunction with the XRD and XRF results, confirm the successful removal of framework Al, which serves as a prerequisite for the formation of structural vacancies and silanol (Si–OH) defects.

To directly confirm the formation of silanol defects in the dealuminated zeolites, DRIFTS measurements were performed, and the spectra in the ca. 3500 cm^−1^ region are shown in Figure 8B. The absorption band in the 3400–3600 cm^−1^ range is attributed to internally H-bonded silanol nests [23]. As summarized in Figure 8C, the relative intensity (*I*/*I*_0_) was defined as the silanol signal intensity relative to that of the parent Y zeolite, where *I* and *I*_0_ are the silanol peak intensities in DRIFT spectra of the modified and parent zeolites, respectively. The *I*/*I*_0_ values for Y-DTPA-0.01 and Y-DTPA-0.1 are 1.50 and 1.75, respectively, both higher than that of Y-parent (1.00), confirming the successful introduction of silanol nest defects, and their concentrations increase with an increase in DTPA concentration used for dealumination. After Pt loading, the silanol-related signal intensity in DRIFT spectra markedly decreases. As for Y-DTPA-0.01-Pt, the *I*/*I*_0_ drops to 1.05, nearly equivalent to the parent zeolite, indicating that most of the internal silanol nests were consumed through strong interaction with dispersed Pt^2+^. In the case of Y-DTPA-0.1-Pt, the *I*/*I*_0_ remains at 1.28, implying that although some silanol defects were utilized to anchor Pt, a significant portion remained unoccupied. These observations are in line prior studies proposing that silanol nest defects serve as: (1) anchoring sites for Pt^2+^ ions via strong interactions, stabilizing them as single-atom species and/or (2) nucleation centers for the formation of metallic Pt^0^ clusters [12,23].

**Figure 9:**
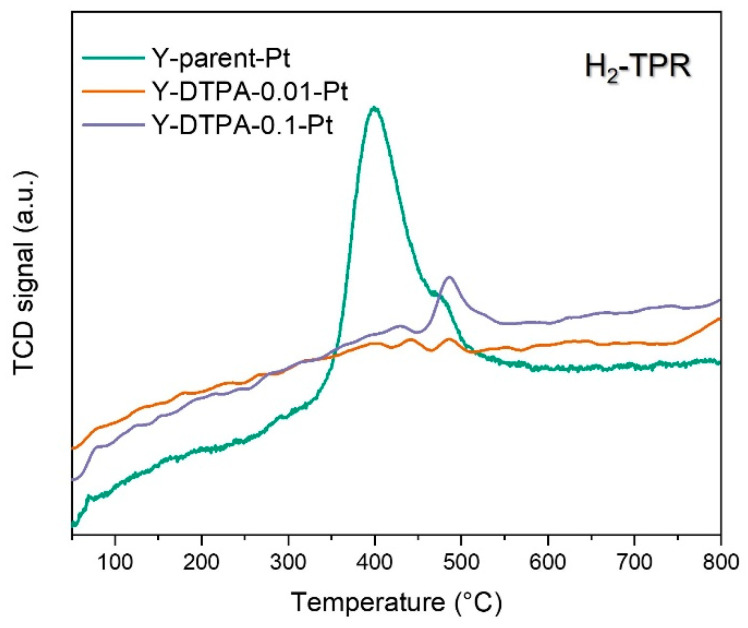
H_2_-TPR profiles of the Pt-loaded Y zeolites.

To further elucidate the status of Pt species anchored at defect sites in Y zeolite, H_2_-TPR analysis was conducted, and the corresponding reduction profiles of Pt-loaded Y zeolites are shown in Figure 9. As for the Y-parent-Pt catalyst using the parent Y zeolite as support, a considerable H_2_ consumption peak centered around 400 °C is observed, indicating the presence of reducible Pt oxides, such as PtO. This observation aligns with the lack of framework defects or anchoring sites in the parent Y zeolite, which limits the stabilization of metallic Pt^0^ clusters or isolated Pt^2+^ anchored in silanol nests). During calcination, Pt precursors without strong interaction with the support tend to aggregate and oxidize in air, leading to PtO formation in Y-parent-Pt. Although PtO has catalytic activity for toluene oxidation, it is generally considered less effective than metallic Pt^0^. In contrast, both Y-DTPA-0.01-Pt and Y-DTPA-0.1-Pt samples exhibit very weak H_2_-TPR signals, suggesting an almost absence of oxidized PtO species. The Pt^2+^ detected by XPS in Y-DTPA-0.01-Pt can thus be attributed to isolated Pt^2+^ ions strongly anchored at silanol nests or hydroxylated vacancies, i.e., defect sites introduced via DTPA-mediated dealumination, which stabilize the Pt species in a highly dispersed state. These strongly anchored Pt^2+^ can be reflected in XPS but with nearly no reducibility in H_2_ gas flow, even at temperature high to 500 °C.

For the support treated with a higher concentration of DTPA (Y-DTPA-0.1), more severe dealumination occurs, resulting in a wider range of internal defects [24]. It is likely that multiple neighboring Al sites are simultaneously removed, leading to the formation of larger defect vacancy clusters. These oversized vacancies hinder the anchoring of Pt as isolated Pt^2+^ species. Instead, such large defect sites may provide sufficient space for the aggregation and reduction of Pt, facilitating the formation of nanoscale metallic Pt^0^ clusters adjacent to the vacancies during high-temperature calcination. This explains the highest Pt^0^/Pt^2+^ ratio observed for Y-DTPA-0.1-Pt. In summary, the Pt species on Y-DTPA-0.01-Pt exist as a well-balanced mixture of Pt^0^ nanoparticles and highly dispersed Pt^2+^anchored in Si–OH defect nests, rather than forming PtO with inferior catalytic activity. Combined with the highest Pt loading enabled by the well-preserved pore structure of its support, this balance between Pt^0^ nanoparticles and defect-stabilized Pt^2+^ underlies the superior catalytic performance for toluene oxidation.

## 4. Conclusions

In this study, a mild and controllable post-synthetic modification using DTPA was developed to tailor framework defects in Beta and Y zeolites, aiming to improve VOC adsorption and catalytic oxidation. By adjusting DTPA concentration (0.01 M and 0.1 M), the degree of dealumination and structural changes were finely controlled. For Y zeolite, the SAR increased significantly from 2.6 to 5.2 after 0.1 M DTPA treatment, indicating substantial Al extraction. This modification enhanced toluene adsorption capacity from 59 to 110 mg/g due to decreased polarity and better pore accessibility. After Pt loading, both Beta- and Y-based catalysts from DTPA-treated supports showed superior catalytic oxidation performance. Notably, Y-DTPA-0.01-Pt achieved 90% toluene conversion at 150 °C with CO_2_ selectivity over 90%, outperforming all other Beta and Y zeolite catalysts prepared in this study. This is attributed to the highest Pt loading (0.72 at%) and a moderate Pt^0^/Pt^2+^ ratio, reflecting efficient Pt dispersion in active forms. Characterizations revealed that dealumination generated internal silanol (Si–OH) nests, confirmed by DRIFTS with relative intensity increasing to 1.50 and 1.75 for 0.01 M and 0.1 M treatments. These nests served as anchoring sites for Pt^2+^, shown by silanol signal decrease after Pt loading (Relative intensity of hydroxyl groups *I*/*I*_0_ down to 1.05 for Y-DTPA-0.01-Pt). XPS indicated a balanced Pt^0^/Pt^2+^ state, while H_2_-TPR showed no reducible PtO species, suggesting strong Pt^2+^-defect interaction. For Y-DTPA-0.01-Pt, nearly complete silanol consumption implied highly dispersed atomic-scale Pt anchoring, contributing to optimal redox and catalytic properties. In summary, this work offers a practical defect engineering strategy via mild chelation to enhance VOC adsorption and catalytic oxidation, providing insights for designing advanced zeolite-supported catalysts for low-temperature VOC abatement.

## Figures and Tables

**Table 1:** Pore parameters of the Beta and Y zeolites.

Sample	*S*_BET_ ^a^ (m^2^/g)	*V*_t_ (cm^3^/g)	*S*_mic_ ^b^ (m^2^/g)	*V*_mic_ ^b^ (cm^3^/g)	*D*_p_ (nm)	*V*_mic_/*V_t_*^c^ (-)	SAR ^d^
β-parent	670	0.38	614	0.24	2.28	0.63	12.5
β-DTPA-0.01	609	0.43	572	0.23	2.80	0.53	17.5
β-DTPA-0.1	496	0.39	457	0.18	3.12	0.47	23.5
Y-parent	810	0.35	793	0.30	1.74	0.86	2.6
Y-DTPA-0.01	827	0.37	796	0.30	1.80	0.81	3.4
Y-DTPA-0.1	670	0.32	638	0.25	1.92	0.76	5.7

^a^ Calculated using the multi-point BET method. ^b^ Calculated using the t-plot method. ^c^ Pore hierarchy, defined as the ratio of micropore volume to total pore volume. ^d^ Calculated based on XRF data.

**Table 2:** Fitting parameters of pseudo-first-order and pseudo-second-order model.

Sample	Pseudo-First-Order-Model		Pseudo-Second-Order-Model	
	*K*_1_	*R* ^2^	*K*_2_	*R* ^2^
β-parent-Pt	0.0122	0.997	1.3573 × 10^−4^	0.955
β-DTPA-0.01-Pt	0.0164	0.995	2.4641 × 10^−4^	0.943
β- DTPA-0.1-Pt	0.0194	0.999	4.1845 × 10^−4^	0.927
Y-parent-Pt	0.0074	0.998	1.1106 × 10^−4^	0.974
Y- DTPA-0.01-Pt	0.0081	0.998	9.1594 × 10^−5^	0.975
Y- DTPA-0.1-Pt	0.0123	0.998	1.5198 × 10^−4^	0.938

**Table 3:** Pt content and valence states measured by XPS.

Sample	Pt Content (at%)	Pt^0^/Pt^+2^
β-parent-Pt	0.18	0.47
β-DTPA-0.01-Pt	0.21	0.50
β-DTPA-0.1-Pt	0.13	1.14
Y-parent-Pt	0.63	0.69
Y-DTPA-0.01-Pt	0.72	0.70
Y-DTPA-0.1-Pt	0.34	0.98

## Data Availability

The datasets used and/or analysed during the current study available from the corresponding author Z.Q. on reasonable request via qiezhipeng@bjut.edu.cn.

## Supplementary Materials

The following supporting information can be downloaded at: https://www.mdpi.com/article/10.3390/toxics13090737/s1. Figure S1 Fitting results of toluene adsorption capacity versus time of the sample (A) β-parent, (B) β-DTPA-0.01, (C) β-DTPA-0.1, (D) Y-parent, (E) Y-DTPA-0.01 and (F) Y-DTPA-0.1. Figure S2 XPS survey spectra of the sample (A) β-parent, (B) β-DTPA-0.01, (C) β-DTPA-0.1, (D) Y-parent, (E) Y-DTPA-0.01 and (F) Y-DTPA-0.1. Table S1 Acidic properties of the Y zeolite catalysts measured by NH_3_-TPD. Table S2 Comparison of different zeolite modification strategies for Pt-loaded catalysts in toluene oxidation.
