# Supplementary material for: Moderate Dealumination of Zeolites via Chelation to Facilitate Pt Anchoring and Toluene Removal
Source: Toxics. 2025 Aug 31;13(9):737. doi: 10.3390/toxics13090737 (PMC12473900; doi:10.3390/toxics13090737)
Supplement: Supplementary file 1 [file toxics-13-00737-s001.zip › toxics-3831300-supplementary.pdf]

Supplementary Material of

Moderate Dealumination of Zeolites via Chelation to Facilitate Pt Anchoring and Toluene Removal

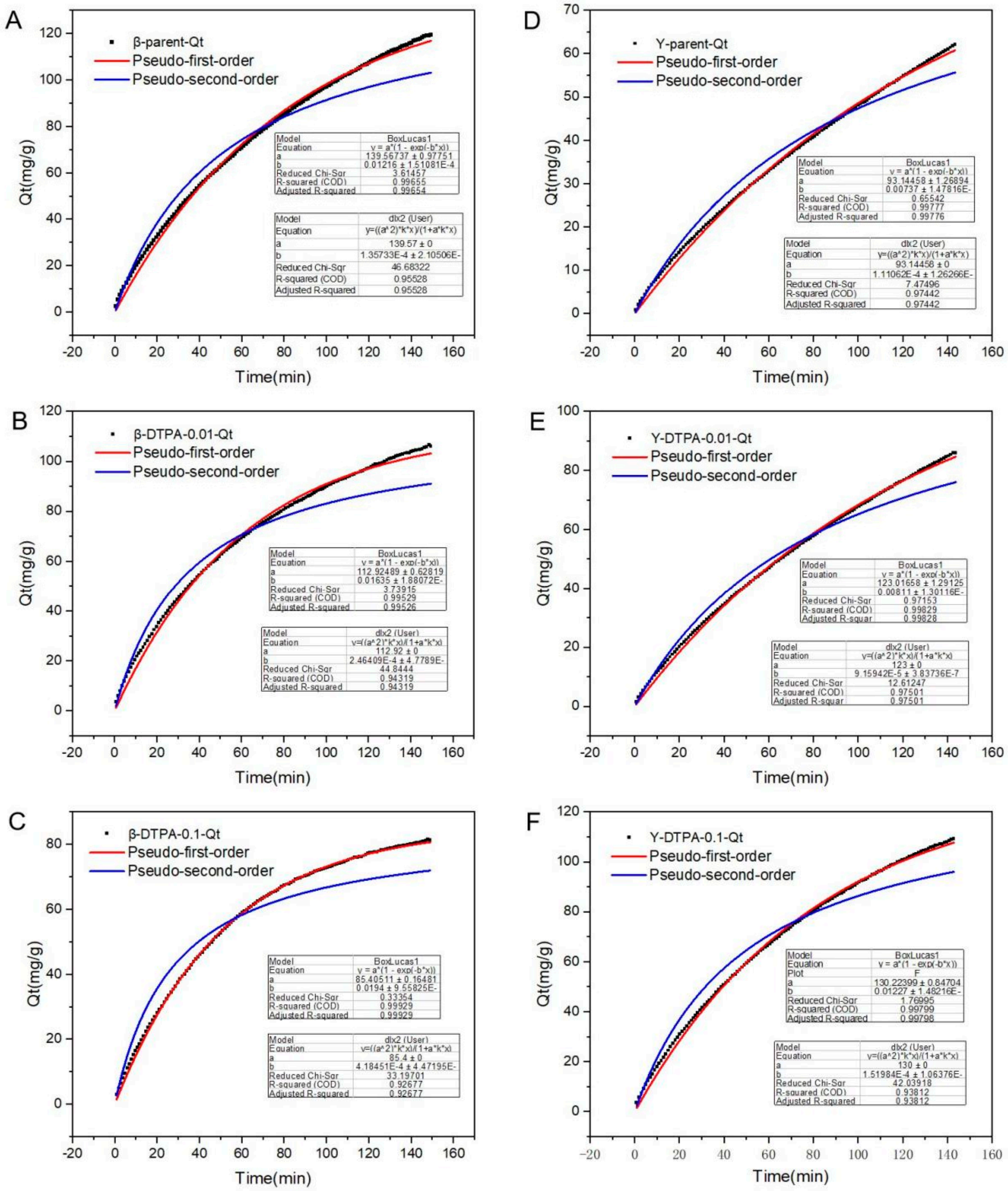

Figure S1 Fitting results of toluene adsorption capacity versus time of the sample (A) β-parent, (B) β-DTPA-0.01, (C) β-DTPA-0.1, (D) Y-parent, (E) Y-DTPA-0.01 and (F) Y-DTPA-0.1

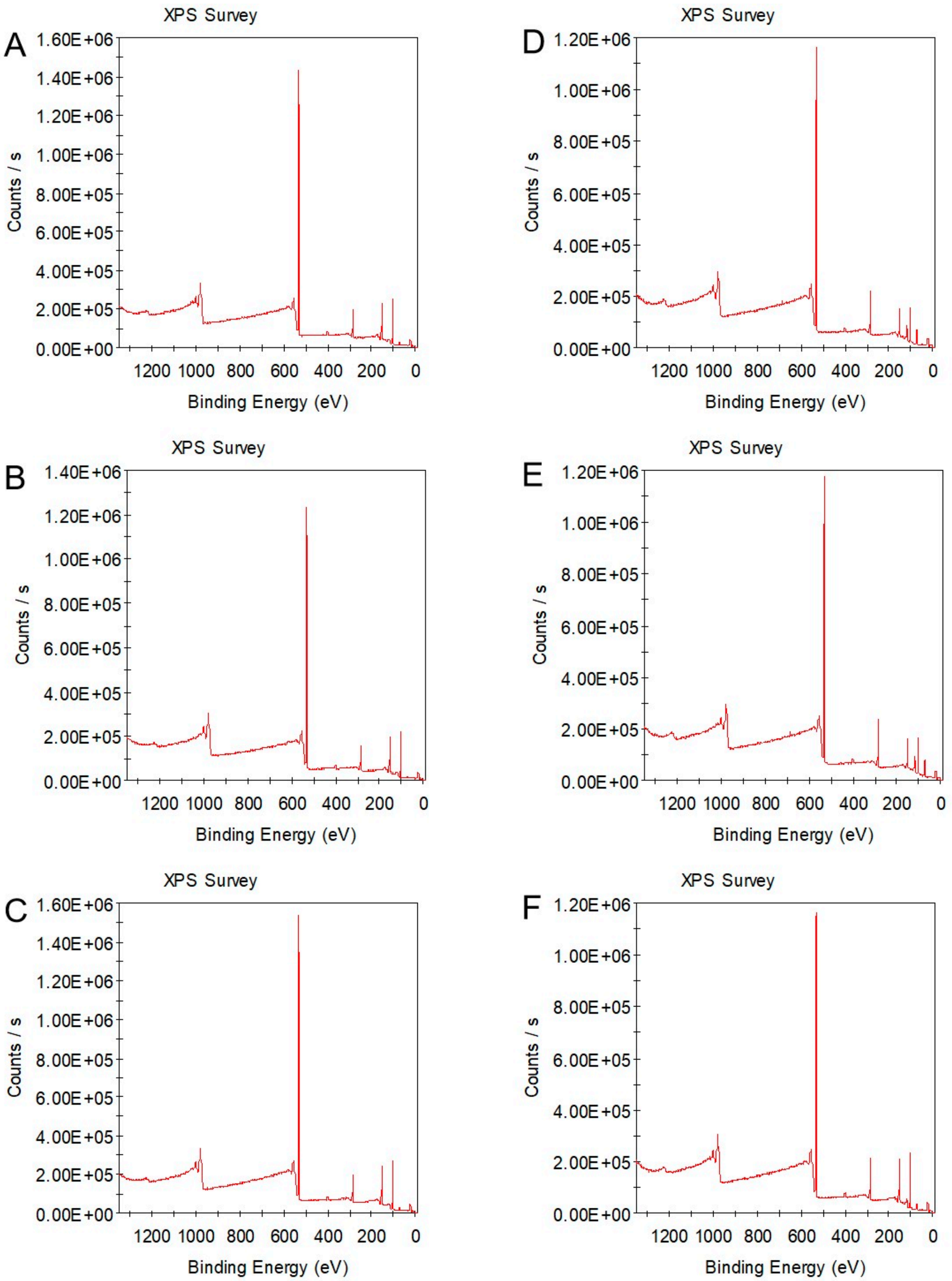

Figure S2 XPS survey spectra of the sample (A) β-parent, (B) β-DTPA-0.01, (C) β-DTPA-0.1, (D) Y-parent, (E) Y-DTPA-0.01 and (F) Y-DTPA-0.1

Table S1 Comparison of different zeolite modification strategies for Pt-loaded catalysts in toluene oxidation.

Catalyst	Post-treatment method	BET surface area (m^2/g)	T_{90} for toluene oxidation (°C)	Reference
Beta-DTPA-0.01-Pt	DTPA chelation	640	~160	This study
Y-DTPA-0.01-Pt	DTPA chelation	720	~150	This study
Pt/deAl-Beta	HNO_3 treatment	395	~190	[12]
Pt/Y-6h	NH_4HF_2 and NH_4OH treatment	480	~149	[22]

Table S2 Acidic properties of the Y zeolite catalysts measured by NH_3-TPD

Sample	Weak acidity (mmol/g)	Strong acidity (mmol/g)	Total acid amount (mmol/g)	Percentage of strong acidity (%)
Y-Parent	0.72	0.55	1.27	43.3
Y-DTPA-0.01	0.43	0.41	0.84	48.8
Y-DTPA-0.01-Pt	0.39	0.35	0.74	47.2
